# Surface roughness prediction of aircraft after coating removal based on optical image and deep learning

**DOI:** 10.1038/s41598-022-24125-5

**Published:** 2022-11-12

**Authors:** Qichun Hu, Haojun Xu, Yipeng Chang

**Affiliations:** Science and Technology on Plasma Dynamics Laboratory, Air Force Engineering University, Xi’an, 710038 Shaanxi China

**Keywords:** Engineering, Aerospace engineering

## Abstract

To quickly evaluate the surface quality of aircraft after coating removal, a surface roughness prediction method based on optical image and deep learning model is proposed. In this paper, the "optical image-surface roughness" data set is constructed, and SSEResNet for regression prediction of surface roughness is designed by using feature fusion method. SSEResNet can effectively extract the detailed features of optical images, and Adam method is used for training optimization. Experiments show that the proposed model outperforms the other seven CNN backbone networks compared. This paper also investigates the effect of four different learning rate decay strategies on model training and prediction performance. The results show that the learning rate decay method of Cosine Annealing with warm restart has the best effect, its test MAE value is 0.245 μm, and the surface roughness prediction results are more consistent with the real value. The work of this paper is of great significance to the removal and repainting of aircraft coatings.

## Introduction

After the coating on the aircraft is removed by laser cleaning or other technologies, it is necessary to evaluate the surface quality to determine whether it meets the conditions and requirements of coating repainting. Surface roughness is the most used parameter to describe the surface micro-geometry, which can be used to evaluate surface quality ^1,2^. However, surface quality is often assessed by visual observation, touch of the cleaned surface of aircraft, and the use of roughness detector. These methods are not accurate and efficient enough to meet the maintenance support needs of advanced aircraft. At present, intelligent, and automatic detection is the mainstream development trend. The realization of intelligent and automatic detection of surface roughness is conducive to the rapid evaluation of surface quality and has important significance and value for the intelligent and automatic process of aircraft coating removal and repainting.

In recent years, there has been a lot of research work on the prediction of surface roughness based on regression analysis, machine vision and neural network. Yang et al. ^3^ proposed a machine vision detection method suitable for predicting the surface roughness of turning, which predicted the surface roughness value by using the ANN network based on the DEA algorithm. Bal Sundaram et al. ^4^ proposed a new method for sub-element edge surface roughness detection based on machine vision, which can measure the turning surface roughness online. Davim et al. ^5^ established a multiple linear regression equation of surface roughness with cutting speed, feed, and depth of cut as independent variables. Ozcelik et al. ^6^ established the first order and second-order prediction models of surface roughness based on the response surface method. Hu et al. ^7^ applied the BP neural network to the prediction modeling of the surface roughness of high-speed milling and verified the model with high prediction accuracy through experiments. Huang et al. ^8^ extracted the eigenvalues of the cutting force signal and proposed an online monitoring method of surface roughness based on the grey theory of bilateral best fitting, which requires less data and requires no training time. Wu et al. ^9^ carried out envelope analysis, statistical calculation, and frequency normalization extraction of vibration signals, and established a surface roughness prediction model through artificial neural network. Although these methods have great advantages in model prediction accuracy, calculation speed, and data volume requirements, the specific performance of the model depends on empirical processing and the selected algorithm. Neural network and machine learning methods are mainly applied to feature extraction and parameter optimization. The roughness prediction is still obtained by physical calculation, and the end-to-end direct prediction is not realized, so the research of intelligent measurement needs to be further in-depth.

Deep learning is a very popular deep network learning method, which can achieve many end-to-end tasks and has a wide range of applications in the field of computer vision ^10–12^. Levi and Hassncer ^13^ designed a convolutional neural network (CNN), after training, the model can determine the gender and directly predict the age after inputting a face image. Liang et al. ^14^ constructed SCUT-FBP5500 facial aesthetics data set and realized the prediction of the beauty value of facial images by improving three CNN models: AlexNet ^15^, ResNet18 ^16,17^ and ResNet50 ^16,18^. Inspired by these works, we propose to directly predict the surface roughness of the corresponding image position based on optical image and deep learning model, to realize the end-to-end surface roughness prediction. The main contributions are as following:

1. The "optical image-surface roughness" dataset is constructed, and the SSEResNet regression prediction model for directly predicting surface roughness through optical images is proposed.
2. Compared with other CNN models, our proposed method achieved the best results.
3. The effects of different learning rate decay strategies on model training and prediction are studied.

## Construction and preprocessing of dataset

### Data collection

**Figure 1:**
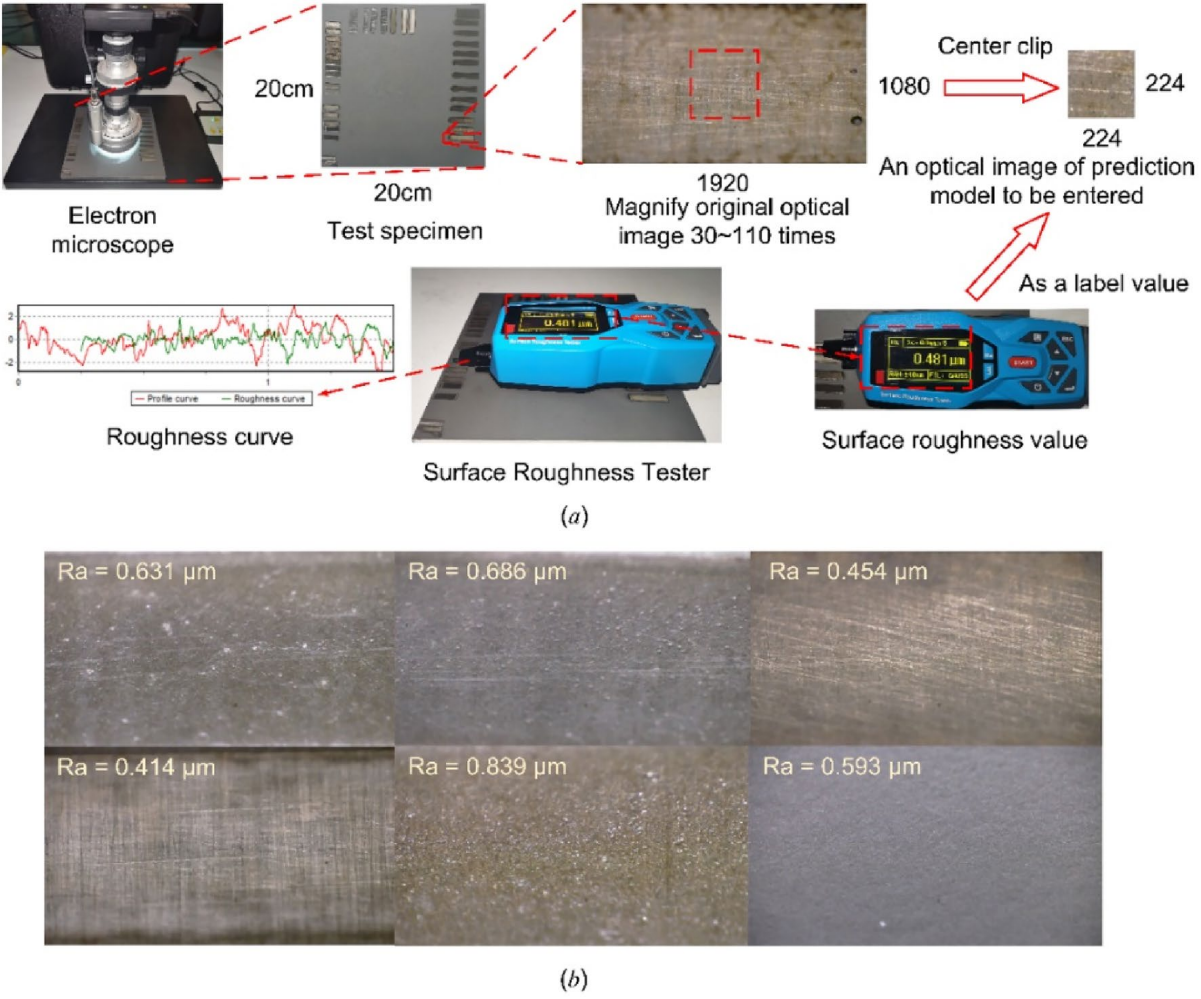
(**a**) Dataset construction process. (**b**) Partial original data.

We sprayed a 20 cm × 20 cm × 1 cm aluminum alloy plate with aircraft special paint, and simulated the removal process of aircraft surface coating by laser cleaning instrument to obtain the experimental plate for collecting surface roughness. We acquired optical images of the experimental plate surface by electron microscopy with random magnification of 30–110 times, and ensured that these optical images contained good surface features. The aspect ratio of the optical image is 1920:1080. In order to meet the input settings of the model, the collected original image is trimmed in the center to obtain the optical image with the aspect ratio of 224:224. Then the roughness value of the corresponding position is measured by the surface roughness tester and used as the label value of the corresponding optical image. And we made the "optical image-surface roughness" regression prediction dataset. In this paper, a total of 10,000 optical images were collected and the surface roughness of 10,000 corresponding positions was measured. Figure 1 shows the dataset construction process and part of the original data.

### Data augmentation

**Figure 2:**
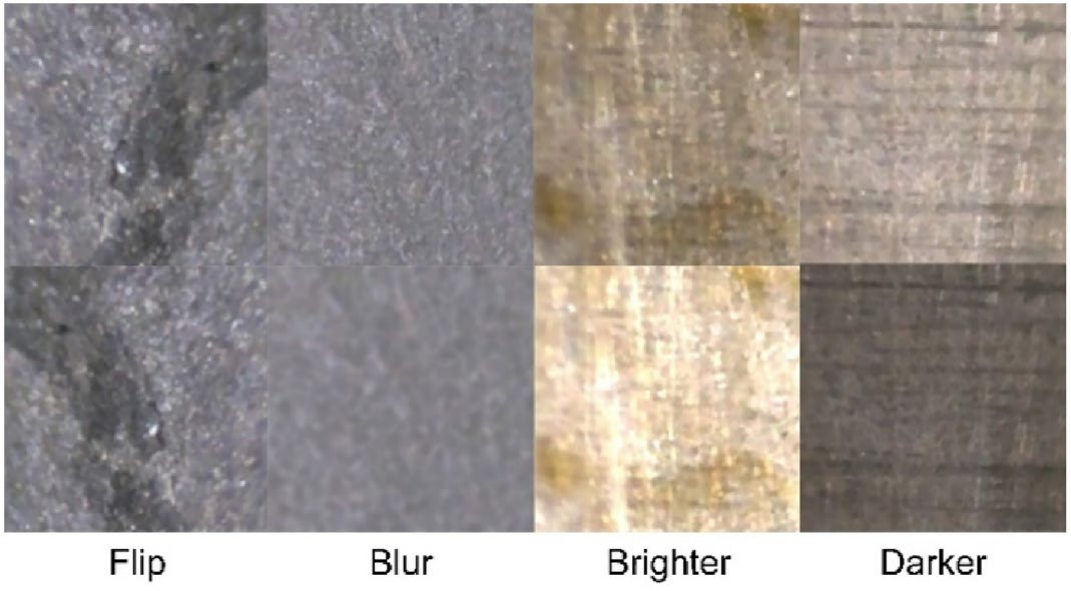
Data augmentation methods.

Deep learning models can learn task-related knowledge from a large amount of data through training. In order to increase the amount of data, we use data enhancement technology to expand the existing datasets. Data augmentation is one of the most important machine learning methods, which is to generate more training data based on the existing training sample data. Its purpose is to make the amplified training data as close as possible to the real distributed data, so as to improve the detection accuracy. In addition, data enhancement can force the model to learn more robust features, so as to effectively improve the generalization ability of the model. In this paper, three data enhancement methods are adopted: flip, brightness change and blur. Flip is to reverse the image, which can preserve the image features while weakening the influence of special positions on image features. Brightness change is to make the image darker or brighter by switching the image to HSL channel and adjusting the L parameter. Blurring is to reduce the image resolution. Figure 2 shows the above data augmentation methods. After the original data is enhanced, the aspect ratio of the obtained image is 224:224, and the amount of data has increased four times.

## Deep learning methods

### Design of "optical image-surface roughness" regression prediction model

**Figure 3:**
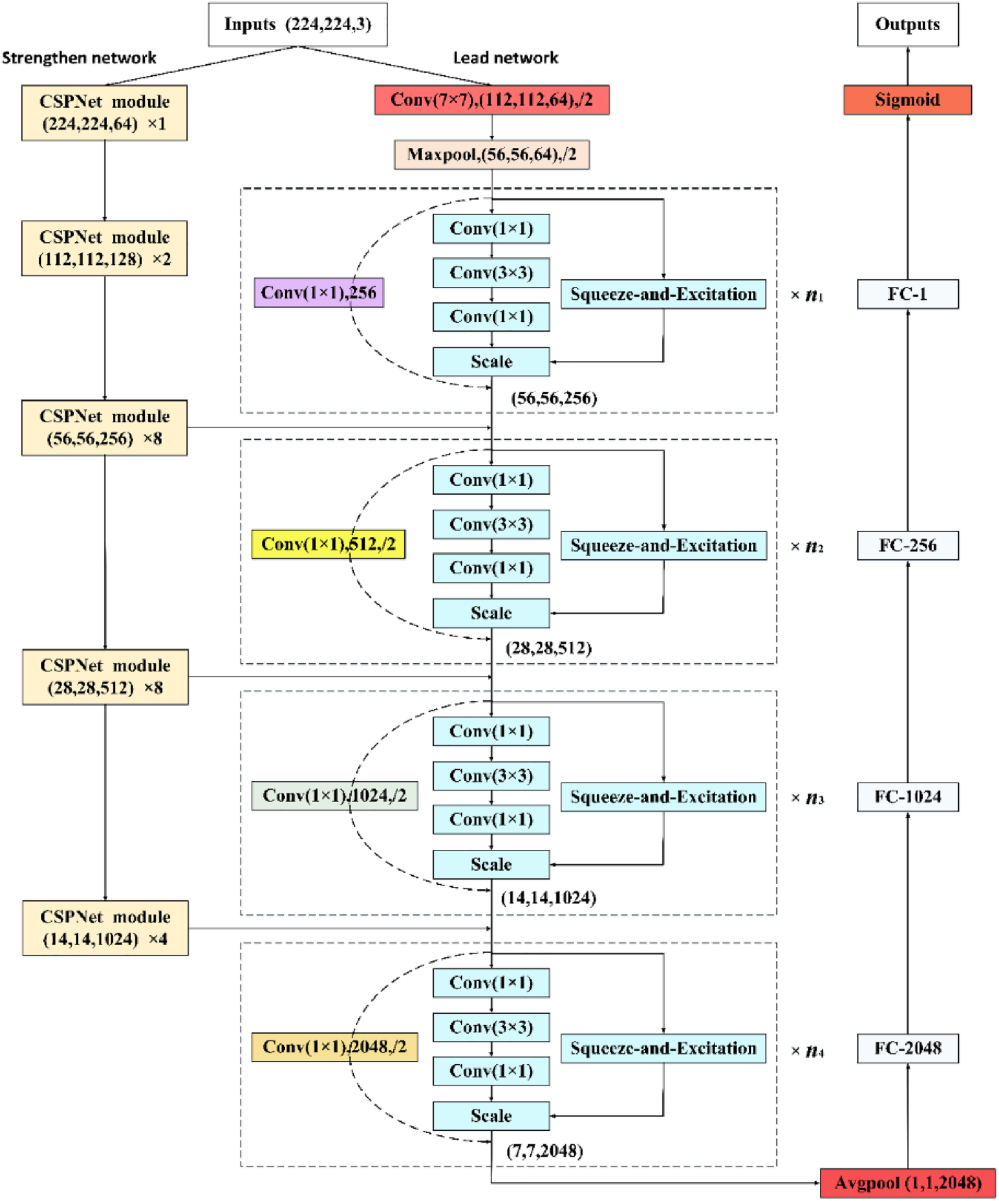
The structure of SSEResNet regression model.

The "optical image-surface roughness" dataset constructed in this paper has the characteristics of large brightness variation and small and complex texture features. What we study is not the classification problem, but the direct prediction of surface roughness through optical images. This is a regression problem, which has higher requirements on the feature extraction ability of the CNN model, and a simple CNN model cannot meet our task requirements. Therefore, we design a CNN model for image regression prediction based on ResNet ^16^, which can better extract complex details and multi-level semantic information from images and achieve the prediction of surface roughness value through optical images. We call it SSEResNet regression model. According to the parameters of ResNet structure used in the model, there are SSEResNet50, SSEResNet101 and SSEResNet152, as shown in Table 1. SSEResNet regression model consists of two parts, the lead network and the strengthen network. The model is shown in Fig. 3.

**Table 1:** SSEResNet regression model parameters.

	*n*_1_	*n*_2_	*n*_3_	*n*_4_
SSEResNet50	3	4	6	3
SSEResNet101	3	4	23	3
SSEResNet152	3	8	36	3

The lead network contains the ResNet backbone and the Squeeze-and-Excitation (SE ^19^) module. The strength network consists of the Cross Stage Partial Network (CSPNet ^20^) module stack. SSEResNet fuses feature maps output by the 3rd, 4th and 5th CSPNet modules with the feature maps output by the 1st, 2nd and 3rd modules of lead network. The feature maps output by each module in the lead network contains relatively low-level semantic information, while the CSPNet module in the strengthen network can extract feature maps containing higher-level semantic information. Through the fusion of high-level features and low-level features, feature maps containing richer semantic information can be generated. Equation (1) expresses the fusion process of feature maps.

1 $$F_{f} = W\left( {F_{l} * F_{s} } \right)$$

Among them, * represents feature fusion, the specific operation is to superimpose the feature map on the channel dimension. *F*_*f*_ represents the feature map after fusion, *F*_*l*_ represents the feature map output by the lead network, *F*_*s*_ represents the feature map output by the strengthen network, and W is a 1 × 1 convolution operation to adjust the channel dimension.

**Figure 4:**
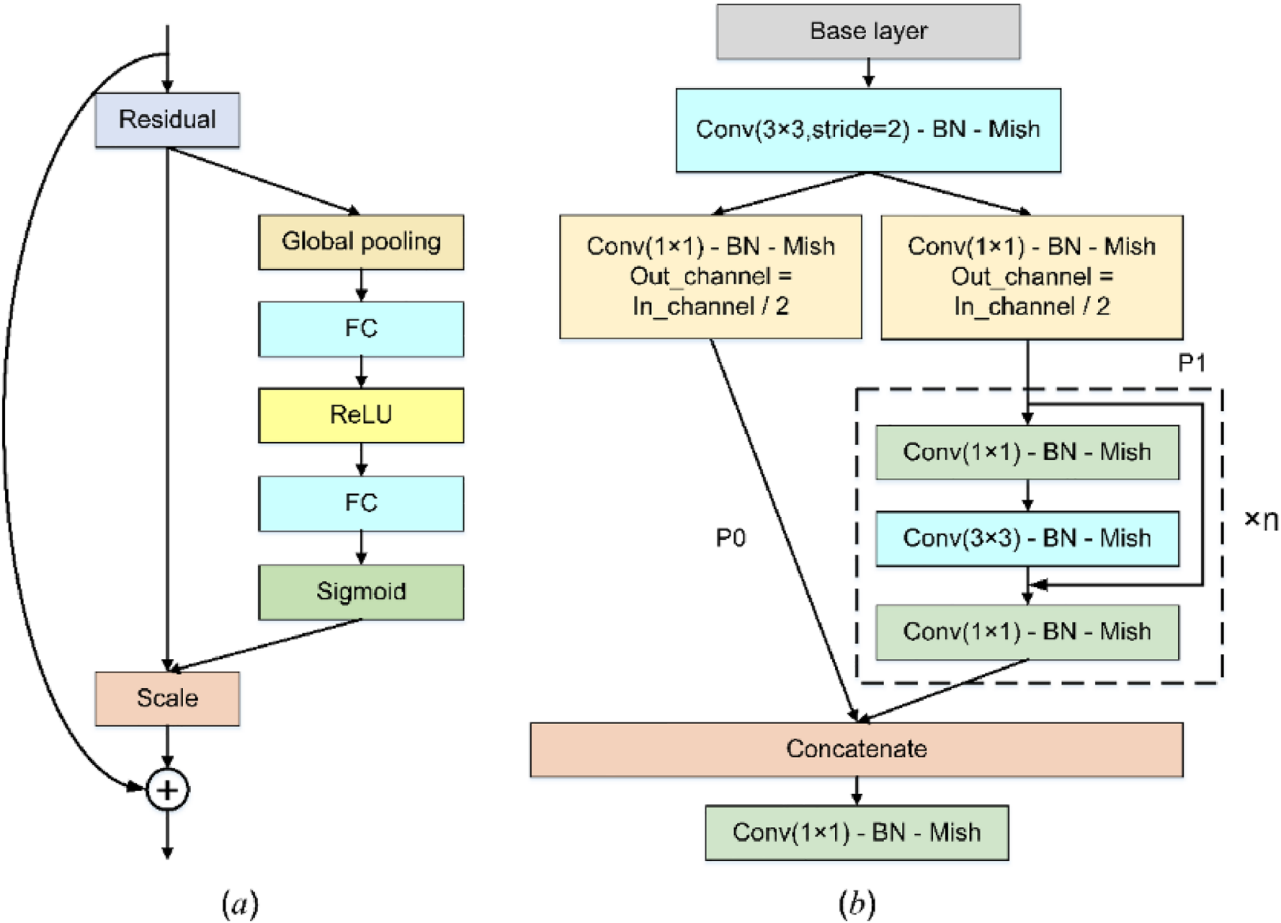
The structure of SE and CSPNet module. (**a**) SE module. (**b**) CSPNet module.

The structure of SE module is shown in Fig. 4a. The feature map input to SE module firstly compresses the features along the spatial dimension through a global average pooling layer, changing each two-dimensional feature channel into a real number, then reducing the feature dimension through a fully connected layer, and upgrading the feature dimension to the original dimension through a fully connected layer after ReLu activation. These three layers form a Bottleneck structure, which can model the correlation between channels. Then the normalized weight is obtained by Sigmoid operation, and finally the weight is weighted to the features of each channel by a Scale operation.

The structure of CSPNet module is shown in Fig. 4b. The feature map input to CSPNet module is first down sampled by a 3 × 3 convolution with a stride of 2, and then integrated into P0 and P1 by a 1 × 1 convolution operation. P1 is superimposed with P0 after passing through n Resblock modules with Bottleneck structure. Finally, the superimposed feature map is further adjusted through a 1 × 1 convolution layer.

In this paper, the number of output channels is set to 1, and a regression model with output real value can be obtained through network training, which can be used to predict the surface roughness of the input optical image. We use a sigmoid function to normalize the output of the fully connected layer and convert the predicted value of the output to [0,1] interval. The sigmoid function value is calculated by Eq. (2). Where *x* is the output of the fully connected layer.

2 $$f(x) = \frac{{1}}{{{1 + }e^{ - x} }}$$

### Model training strategy

#### Adam optimization algorithm

In this paper, Adam optimization algorithm is used to optimize the model in the training process. ADAM ^21^ (Adaptive Moment Estimation) is a first-order optimization algorithm that can replace the traditional stochastic gradient descent process. It can iteratively update the weights of the network based on the training data, which is essentially RMSprop ^22^ with a momentum term. The learning rate of each parameter is dynamically adjusted by using the first moment estimation and the second moment estimation of the gradient. Its advantage is that after offset correction, the learning rate of each iteration has a certain range, which makes the parameter change more stable. Equation (3) expresses the calculation method of Adam.

3 $$\left\{ {\begin{array}{*{20}c} {\Delta \theta_{t} = - \alpha \frac{{\hat{m}_{t} }}{{\sqrt {\hat{n}_{t} } + \varepsilon }}} \\ {\hat{m}_{t} = \frac{{m_{t} }}{{1 - \beta_{1}^{t} }},\hat{n}_{t} = \frac{{n_{t} }}{{1 - \beta_{2}^{t} }}} \\ {m_{t} = \beta_{1} m_{t - 1} + \left( {1 - \beta_{1} } \right)\frac{\partial L}{{\partial \theta_{t} }}} \\ {n_{t} = \beta_{2} n_{t - 1} + \left( {1 - \beta_{2} } \right)\left( {\frac{\partial L}{{\partial \theta_{t} }}} \right)^{2} } \\ \end{array} } \right.$$

Among them, the last two formulas are the first-order moment estimation and the second-order moment estimation of the gradient, which can be dynamically adjusted according to the gradient. The formula in the second line is the correction of the moment estimation, which can be approximated as an unbiased estimation of the expectation. The first formula is a dynamic constraint on the learning rate *α*, with a clear range. Parameter *t* is the number of iterations, *ε* can keep the denominator from being zero, $\varepsilon = 1 \times 10^{ - 9}$, $\beta_{1} = 0.9$,$\beta_{2} = 0.999$.

#### Learning rate decay method

In this paper, the learning rate decay method is used to iteratively train the model, mainly including equal interval adjusted learning rate (StepLR), given interval adjusted learning rate (MultiStepLR), cosine periodic adjusted learning rate (CosineAnnealingLR) and CosineAnnealing with warm restart。

StepLR sets the learning rate of each parameter group to the initial learning rate decayed by gamma every step size epoch. We set step size to 30, gamma = 0.1.

MultiStepLR can be manually set in which epochs with gamma as the adjustment coefficient to decay the learning rate. In this paper, the 30th and 80th epochs are set to decay the learning rate with gamma = 0.1.

**Figure 5:**
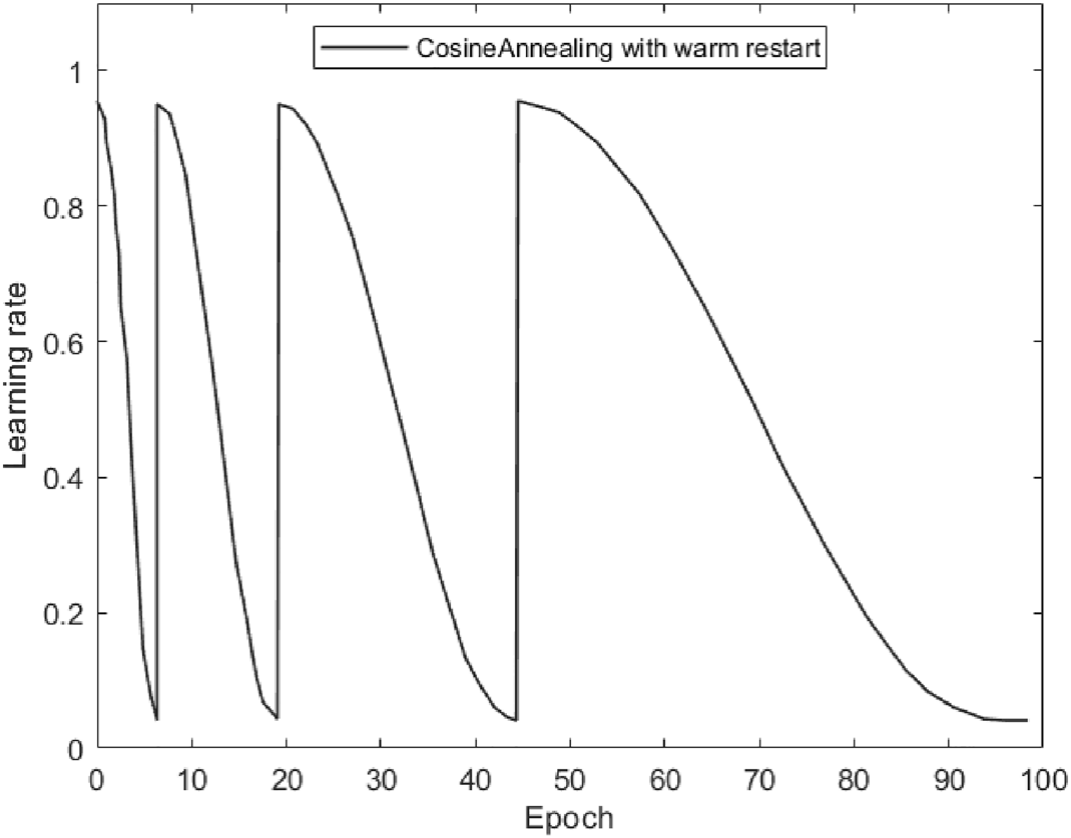
The change trend of learning rate using cosine annealing with warm restart.

CosineAnnealingLR uses the cosine method to decay the learning rate. The decay process is like the cosine function. Equation (4) is its calculation method, where *T*_max_ is the maximum decline period. The introduction of the warm restart operation can make the change of the learning rate no longer simply decrease but have a certain fluctuation. The changing trend of the learning rate is shown in Fig. 5.

4 $$\alpha_{t} = \alpha_{\min } + \frac{1}{2}\left( {\alpha_{\max } - \alpha_{\min } } \right)\left( {1 + \cos \left( {\frac{{T_{current} }}{{T_{\max } }}\pi } \right)} \right)$$

## Experiments and Results

### Experimental environment and parameter settings

Table 2 shows the experimental environment of this paper. Batch size = 32, Max epoch = 100, and the weight of the last epoch is taken as the model training result. When using Adam optimization algorithm, learning rate = 0.001, momentum = 0.9.

**Table 2:** Experimental environment.

Operating system	Graphics card	PyTorch	CUDA
Windows 10	Nvidia GTX 2080	1.1	10.0

### Comparative experiments of different models

In this paper, the prediction performance of three SSEResNet regression models on three datasets of different sizes by using a simple gradient descent (GD) optimization algorithm is compared first. The experimental conditions are the original dataset without data enhancement, a fixed learning rate of 0.0025, 100 epochs and other same parameter configurations. In this paper, mean square error (MSE) loss is used to replace the previous cross-entropy loss used for classification tasks, as an evaluation index of the experiment. MSE is suitable for regression tasks and is calculated by Eq. (5). Where *y*_*i*_ represents the true value and *p*_*i*_ represents the predicted value. Table 3 shows the experimental results of different SSEResNet regression models on different datasets.

5 $$MSE = \frac{1}{n}\sum\limits_{i = 1}^{n} {\left( {p_{i} - y_{i} } \right)^{2} }$$

**Table 3:** Experimental results of different SSEResNet on different datasets.

	SSEResNet50	SSEResNet101	SSEResNet152
6000 images	0.0361	0.0365	0.0382
8000 images	0.0375	0.0354	0.0368
10,000 images	0.0391	0.0362	0.0357

From the experimental data in Table 3, it can be seen that the model needs to match the dataset of appropriate size to achieve good results, and the deeper the network layer, the larger the dataset is needed for the model. This is because the shallow model has insufficient feature extraction and limited image processing ability on large datasets, while the deep model is easy to over-fit on small datasets. Considering the training time and the prediction performance of the model, the SSEResNet101 regression model and the dataset of 8000 images were selected in this paper for the subsequent comparison experiment with other models.

In this paper, four optimization methods are compared, and then the SSEResNet101 model is compared with another seven CNN backbone networks. Using SSEResNet101 regression model, the Adam optimization method is tested under the same conditions as other three optimization methods commonly used in deep learning, which are SGD, Momentum and RMSprop. The experimental conditions are a fixed learning rate of 0.0025, 100 epochs and other identical parameter configurations. MSE loss, mean absolute error (MAE) and R-Square (R2) are selected as the evaluation indexes. Equations (6) and (7) are the calculation methods of MAE and R2, respectively. Where $\overline{y}_{i}$ is the mean of the label values. When the predicted value is equal to the label value, MAE is equal to 0, and the greater the error, the greater the MAE value. The value range of R-Squared is [0,1]. If the result is 0, the model fitting effect is poor; if the result is 1, the model is completely fitted. The larger the R-Squared, the better the model fitting effect. Table 4 shows the experimental results of different optimization methods.

6 $$MAE = \frac{1}{n}\sum\limits_{i = 1}^{n} {\left| {p_{i} - y_{i} } \right|}$$

7 $$R2 = 1 - \frac{{\sum\limits_{i} {(p_{i} - y_{i} )^{2} } }}{{\sum\limits_{i} {(\overline{y}_{i} - y_{i} )^{2} } }}$$

**Table 4:** Experimental results of different optimization methods.

optimization methods	Unused data augmentation			Used data augmentation		
	R2	MAE(μm)	Test MSE	R2	MAE(μm)	Test MSE
SGD	0.8936	0.5213	0.0516	0.9071	0.4691	0.0469
Momentum	0.9375	0.3521	0.0378	0.9539	0.3224	0.0324
RMSprop	0.9542	0.3412	0.0362	0.9687	0.3169	0.0318
Adam	0.9789	0.3214	0.0313	0.9861	0.2929	0.0285

The experimental data in Table 4 show that the Adam optimization method has better performance than the other three optimization methods both without and with data enhancement. Compared with Momentum, RMSprop and traditional SGD algorithm, Adam integrates the advantages of Momentum and RMSprop. Among them, the advantage of Momentum is that it can accelerate the learning of parameters with the same gradient direction, and reduce the update of parameters with the change of gradient direction, so that parameters in the same direction can converge quickly. RMSprop is an adaptive learning rate optimization algorithm. The advantage of RMSprop is that in the early training stage, the learning rate is large, which can accelerate the convergence of the model, while in the later training stage, the learning rate is small, which is beneficial to suppress the model oscillation and avoid skipping the optimal solution. Therefore, we use Adam optimization method to conduct a comparative experiment between SSEResNet101 and seven other CNN backbone networks. The experimental conditions are fixed learning rate of 0.0025, 100 epochs and other identical parameter configurations. Table 5 shows the experimental results of the regression prediction models.

**Table 5:** Experimental results of “optical image-surface roughness” regression models.

	The values of three regression evaluation indexes					
	Unused data augmentation			Used data augmentation		
	Test MSE	MAE(μm)	R2	Test MSE	MAE(μm)	R2
VGG16^23^	0.0560	0.5131	0.8850	0.0512	0.4618	0.9110
ResNet50^16,18^	0.0483	0.4452	0.9039	0.0443	0.4023	0.9419
ResNet101^16,24^	0.0410	0.3982	0.9288	0.0382	0.3611	0.9685
ResNet152^16,25^	0.0392	0.3716	0.9500	0.0368	0.3424	0.9734
EfficientNet-B0 ^26^	0.0461	0.4040	0.9327	0.0425	0.3795	0.9510
SEResNet101^19^	0.0346	0.3497	0.9572	0.0317	0.3102	0.9753
CSPDarkNet53^20^	0.0354	0.3511	0.9577	0.0321	0.3140	0.9744
Ours	0.0313	0.3214	0.9789	0.0285	0.2929	0.9861

The experimental data in Table 5 show that the MSE loss and MAE values of the model are reduced after data enhancement, which indicates that the data enhancement operation effectively improves the performance of the model. This is because the data enhancement operation generates many similar but different training samples by making a series of random changes to the training images, thus enlarging the scale of the training dataset. In addition, these random changes make the model less dependent on some attributes in the training samples, thus improving the generalization ability of the model. Compared with other CNN models, the MSE and MAE values of our model are the smallest, and the R2 value is the largest. After using data enhancement, the MSE of our model is only 0.0285, 0.0097 less than ResNet101 and 0.0032 less than SEResNet101. Meanwhile, SEResNet101 was also 0.0065 smaller than ResNet101. These comparison results show that both SE module and CSPNet strengthen module play an important role in improving the prediction ability of the model. This is because the SE module mines the correlation of features among channels, and the CSPNet strengthen module assists in extracting deep semantic information. After feature fusion and mapping with shallow semantic information, richer semantic information can be obtained. Under the joint action of these two modules, the model can better extract the detailed features of optical images, and then learn the mapping relationship between these features and surface roughness.

### Influence of learning rate decay strategy on model training and prediction effect

**Figure 6:**
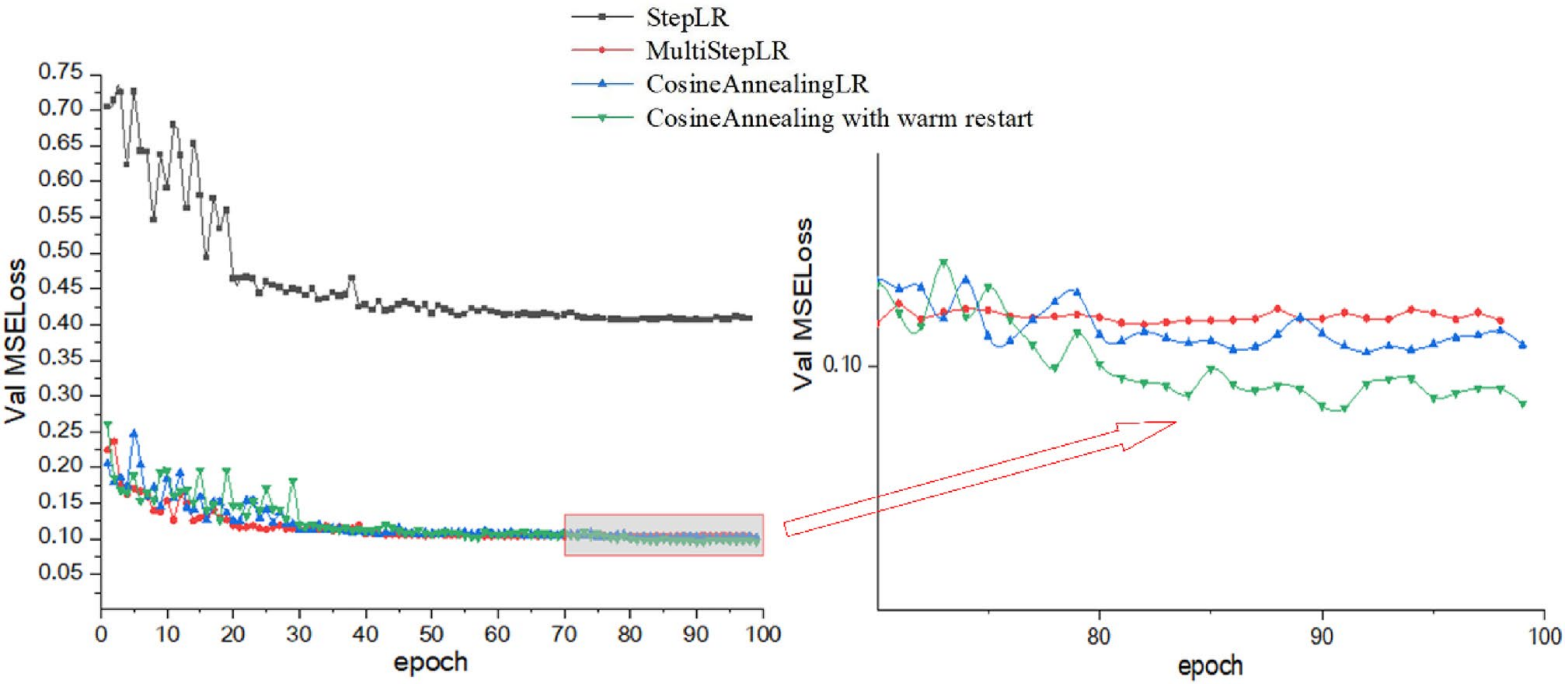
MSE loss curves of validation set for different learning rate decay strategies.

In this paper, we also study the effect of different learning rate decay strategies on the model during training. The MSE loss curves of validation set, which is trained on the dataset of 8000 images by using StepLR, MultiStepLR, CosineAnnealingLR and CosineAnnealing with warm restart, are shown in Fig. 6.

The experimental results in Fig. 6 show that the learning rate attenuation method of CosineAnnealing with warm restart has the best convergence effect, and the MSE loss is the smallest. The CosineAnnealingLR method has the second-best training effect, and StepLR has the worst training effect. CosineAnnealing with warm restart can make the learning rate decline to a certain value, warm restart, return to the initial value, and then conduct a new round of decline. Such a learning rate adjustment method can make the model that converges to the local optimal solution, jump out of the local optimal solution, and continue to update the model until the model reaches the global optimal solution.

**Figure 7:**
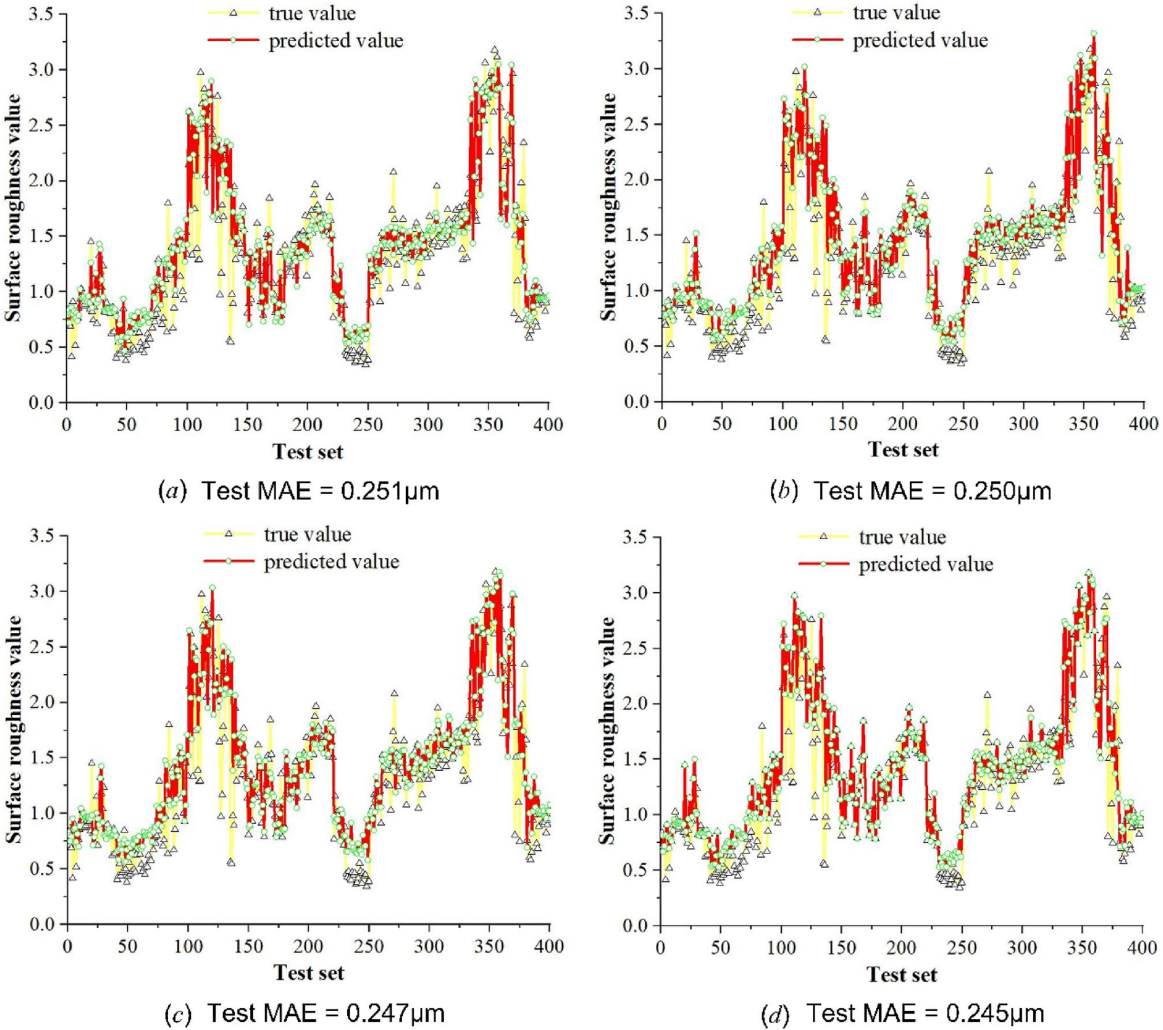
Point plot of predicted and actual surface roughness values of SSEResNet101 regression model. (**a**) Using StepLR. (**b**) Using MultiStepLR. (**c**) Using CosineAnnealingLR. (d) Using CosineAnnealing with warm restart.

To more intuitively show the effect of surface roughness prediction based on optical images and deep learning regression models, we plot the validation results of the test set as a point plot of the predicted values of the regression model and the true label values, as shown in Fig. 7.

The experimental results in Fig. 7 show that the surface roughness predicted by the regression model for optical images is close to the real value, which indicates that the regression model we designed has a good prediction effect and can directly and accurately predict the surface roughness of optical images. Especially, the prediction effect of the learning rate attenuation method of CosineAnnealing with warm restart is the best, its test MAE value is 0.245 μm, and the prediction result of surface roughness is more consistent with the real value.

## Conclusion

In this paper, the prediction of surface roughness of aircraft after coating removal based on optical image and deep learning is studied. First, we use laser cleaning technology to remove the coating of the experimental specimens sprayed with aircraft coatings, and get the experimental specimens used to evaluate the surface roughness. Then, an electronic microscope is used to collect optical images randomly magnified by 30–110 times, and the roughness values of the corresponding optical images are obtained by a surface roughness tester as labels. The dataset of "optical image-surface roughness" is made and three data augmentation methods are used to enhance the data. The experimental results show that the data enhancement operation is effective in improving the performance of the deep learning model. In this paper, the SSEResNet101 regression prediction model is designed by the method of feature fusion, to better extract the detailed features of optical images. The experimental results show that SSEResNet101 has excellent model performance.

We also study the effect of different learning rate decay methods on model training and prediction performance. The results show that the CosineAnnealing with warm restart method has the best training effect and testing effect, the test MAE value is 0.245 μm. The SSEResNet101 regression prediction model designed in this paper can predict the surface roughness directly through optical images, so that it can quickly determine whether the surface with removed coating on the aircraft meets the roughness requirements of repainting.

## Data Availability

Data underlying the results presented in this paper are not publicly available at this time but may be obtained from the corresponding author (email address: kgdhuqichun@163.com).
